# Experimental ultrasound stimulator for improving the diffusion of exosomes and drugs into lung tissue

**DOI:** 10.17221/41/2025-VETMED

**Published:** 2025-11-27

**Authors:** Jaroslav Prucha, Josef Skopalik, Tomas Parak, Petr Bratka, Julie Cuprova

**Affiliations:** ^1^Department of Communication and Information Technologies, Faculty of Biomedical Engineering, Czech Technical University in Prague, Kladno, Czech Republic; ^2^Laboratory Medicine Centre, Trauma Hospital of Brno, Brno, Czech Republic; ^3^Department of Pharmacology and Toxicology, Faculty of Pharmacy, Masaryk University, Brno, Czech Republic; ^4^Department of Healthcare and Population Protection, Faculty of Biomedical Engineering, Czech Technical University in Prague, Kladno, Czech Republic

**Keywords:** drug infusion, electromagnetic stimulus, exosomes, fibrosis, therapeutic ultrasound

## Abstract

Pulmonary fibrosis is not only a consequence of the recent COVID-19 pandemic, but is increasingly recognised by both human and veterinary healthcare providers. Pulmonary fibrosis is a progressive condition that leads to a decline in respiratory function and even death. In this work, we compared MSC-derived exosomes with conventional anti-inflammatory drug treatments. Exosomes from stimulated MSCs displayed higher miRNA concentrations (in particular, miRNA-30b was significantly increased). A set of rats with induced lung fibrosis were divided into four groups: NC (control – no treatment), A2 (exosome infusion), A3 (exosome infusion combined with ultrasound stimulation), and F1 (tamoxifen/metformin drug treatment). The rats’ lungs were subjected to histological analysis; the fibrosis scores for groups F1 and A3 were very similar and decreased significantly compared with group NC. Ultrasound-facilitated diffusion of exosomes from the capillaries into the lung tissue could represent an innovative therapeutic approach for slowing fibrosis and prolonging the active life of the organism.

Exosomes are secreted by various cell types, including mesenchymal stem cells (MSCs) (Yu et al. 2014). Exosomes are small, membrane-bound vesicles that play an essential role in intercellular communication by delivering microRNAs (miRNAs/miRs), mRNAs, and proteins) to recipient cells (Tkach and Thery 2016).

Several studies have demonstrated that exosomes from MSCs can influence the progression of lung fibrosis (Xu et al. 2022). Exosomes derived from MSCs can regulate target genes by delivering functional miRNAs. However, the specific role of exosome-loaded miRNAs in reducing lung inflammation and fibrosis remains unclear.

Among the most frequently studied miRNAs in the context of pulmonary fibrosis treatment are miRNA-30, miRNA-31, and miRNA-150 (Li et al. 2019). Some of these exosomal miRNAs may affect fibroblast transdifferentiation and modulate T-cell subsets (Kuse et al. 2020) or the activation of fibroblasts in the initial phase of fibrosis progression (Niu et al. 2022; Xu et al. 2022; Ding et al. 2023).

In human medicine, particularly following the COVID-19 pandemic, clinical trials have investigated intravenous infusions of whole MSCs to halt the progression of inflammation and organ failure (Barkama et al. 2020; Baig et al. 2024). Although whole-cell infusion or transplantation has been reported to suppress the progression of lung inflammation in many cases, the safety and biodistribution of cell extracts after administration remain uncertain, with concerns regarding the potential risk of microcapillary blockage in the lungs and other organs (Baig et al. 2024). The therapeutic effects of transplanted cells, for instance, on infected lungs during the post-acute phase of COVID-19, have not been clearly demonstrated in either animal models or clinical trials. Therefore, our study aimed to isolate exosomes using a specialised culture system and to administer them intravenously in combination with ultrasound stimulation to accelerate their diffusion from the pulmonary capillaries into the lung interstitium. We compared the effectiveness of treatment according to the parameters of exosome infusion and our original sono-pulse potentiation.

## MATERIAL AND METHODS

### Derivation of exosomes

Mesenchymal stromal cells (MSCs) isolated from rat adipose tissue were washed and cultivated in three CellBIND 25 cm^2^ culture flasks (Corning, Kenee, USA) using DMEM supplemented with 2% foetal bovine serum (FBS) and penicillin/streptomycin (pen/strep) as the culture medium. Further details regarding the isolation and incubation were described by Siciliano et al. (2016). When the cells reached approximately 60% confluence, trypsin-EDTA was used to detach them, and the cells were resuspended and seeded into 48-well culture plates (Corning, Kennee, USA). The same DMEM with 2% FBS and pen/strep was used for subsequent cultivation. The initial cell density was 50 cells per mm^2^. On the sixth day of incubation, the medium was replaced with serum-free medium (400 μl of DMEM with 0.5% penicillin and 5% trehalose per well).

**Figure 1:**
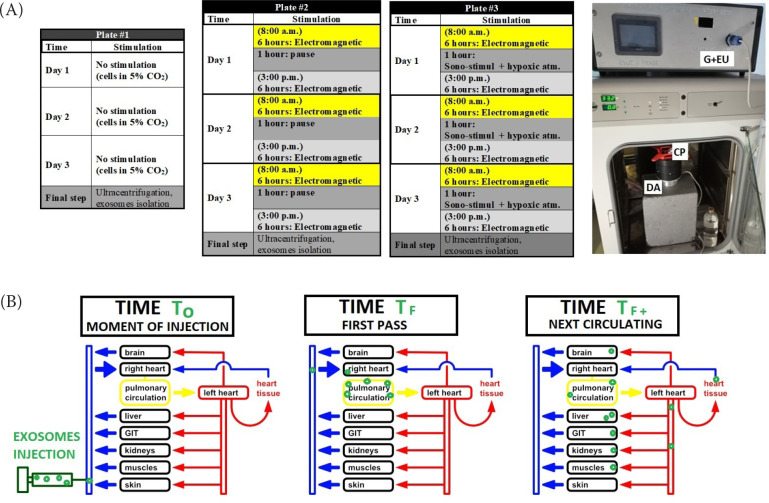
(A) Schematic time schedule for the control culture (Plate #1) and experimental cultures (Plates #2 and #3), and final instrument design of applicators in the incubator The electromagnetic coil generated a basic sinusoidal signal with f = 5 kHz and a modulating signal adapted from Prucha et al. (2019); the induced electrical current density was optimised to 2 A/m^2^ in the culture positioned 2 cm above the coil. (B) Schematic description of exosome or SonoVue particle (green dots) injection into the tail vein and their entrainment into the lungs and other organs over time CP = culture plate; DA = dual applicator consisting of an ultrasound head and electromagnetic coil; G+EU = generator and electronic unit

Three experimental variants of cultivation prior to exosome isolation were prepared and compared. The three culture plates were stimulated under different conditions: Plate #1 was used for simple cultivation (without stimulation by either electromagnetic coils or ultrasound; incubation under normal atmospheric conditions of 5% CO_2_, 21% O_2_); Plate #2 was incubated under the same atmospheric conditions; however, stimulation was applied using the experimental electromagnetic generator KP1000W – CTU Prague (details in Figure 1A); Plate #3 was stimulated in the same way as Plate #2, but with the addition of one hour of hypoxia and an ultrasound stimulus (0.2 W/cm^2^, 1 MHz, with 20/80 ms on/off pulse cycles) between two doses of electromagnetic stimulation (details in Figure 1A). After three days, 12 ml of medium was collected from all variants, and exosomes were isolated using ultracentrifugation.

**Figure 2:**
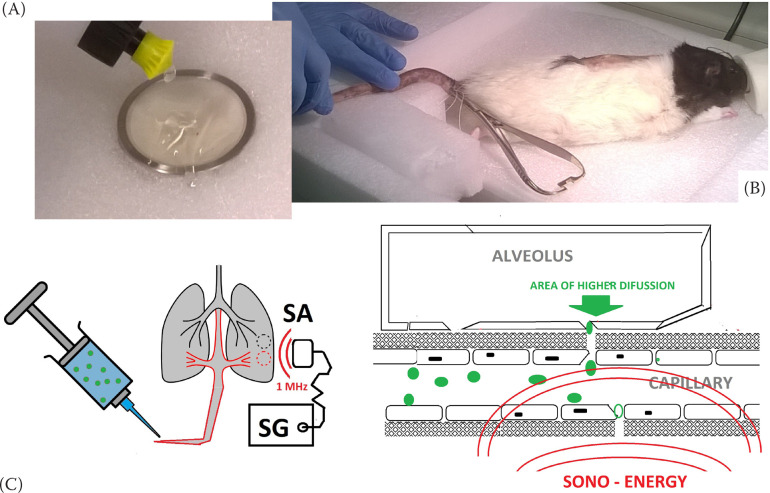
Sono-stimulation (A) Preparation of the pad with the head and contact gel. (B) Placement of the animal on the head in the lung area. (C) Overview of SKP1000 device components and the concept of microscopical principles of endothelial layer permeabilisation by sono-energy SA = sono-applicator; SG = sono-generator

Using real-time PCR, the exosomal miRNA concentration per 1 ml of medium was quantified for all plates (#1, #2, #3). Specifically, the exosomes were stored at −80 °C and thawed in a 37 °C water bath. The total RNA was extracted using the Total RNA Extraction Kit (k.n.8913; Sigma-Aldrich, Darmstadt, Germany) according to the manufacturer’s protocol. The RNA was purified with DNase I to remove DNA contamination, and 0.5 μg of RNA from each sample was used to generate cDNA with the PrimeScript^TM^ RT Reagent Kit (Takara, Shiga, Japan), following the manufacturer’s instructions. The reaction conditions consisted of 40 cycles at 95 °C for 30 s, followed by 95 °C for 5 s, and annealing at 57 °C for 34 seconds. Relative gene expression was calculated using the 2^–ΔΔCt^ method (details in Figure 2).

### Preparation of the lung model

The *in vivo* experiment was conducted at the Research Veterinary Laboratory of the Veteri-nary Research Institute in Brno (Department of Pharmacology and Toxicology). The project was approved by the Ethics Committee of the Ministry of Agriculture of the Czech Republic (Registration No. MZe 2252). Male rats (*Rattus norvegicus*), 5–8 months old, with an initial body weight of 280–350 g, were used. All rats were ZDF homozygous fa/fa strain; this genetic profile leads to the spontaneous development of type 2 diabetes within the first months after birth, depending on the composition of the diet. Because the pathophysiological mechanisms responsible for the development of the disease are similar to those that apply in humans, the ZDF rat is considered a suitable model for studying diabetes-related physiology and treatment responses in secondary lung diseases. Animals were housed in 43 × 28 × 15 cm cages, and water and granules were available *ad libitum*. The experiment began after a 10-day acclimatisation period. Lung fibrosis was induced by bleomycin administration (designated as day 0). Bleomycin was administered by inhalation (inhaled 0.2 U of bleomycin in 4 ml water solution using Omron C101 nebuliser under 3% isoflurane anaesthesia). All animals were monitored closely for 24 h after inhalation.

### Final *in vivo* experimental intervention

Rats were divided into 4 groups: 2 control groups (F1: 10 animals treated with the reference drug metformin; NC: 10 animals without any treatment) and 2 experimental groups (A2, A3; 10 rats in each group). For groups F1, A2, and A3, the administered doses of drugs, exosomes, and physical stimulation protocols were applied as described in Table 1. Exosomes were administered intravenously via the tail vein located 2 cm from the base of the tail. This ensured that most of the exosomes passed primarily through the lung tissue during the first seconds after injection (first-pass effect), minimising their uptake by other organs (for details, see Figure 1B, time T_F_).

**Table 1:** Grouping of animals and experimental treatment procedure

Group	Therapeutic entity administered	Dose	Drug application	Number of animals	Histological examination
F1	tamoxifen metformin	1.5 mg/ml water 0.4 mg/g granules	continuously	10	days 15 and 21
A2	exosomes (extract from MSC)	0.5 mg/kg	days 7 and 10	10	days 15 and 21
A3	exosomes (extract from MSC)	0.5 mg/kg with sono-application 10 W/cm^2^	days 7 and 10	10	days 15 and 21
NC	infusion of phys. saline only	0.5 ml phys. Saline per rat	days 7 and 10	10	days 15 and 21

### Adjustment before final experimental setup

**Figure 3:**
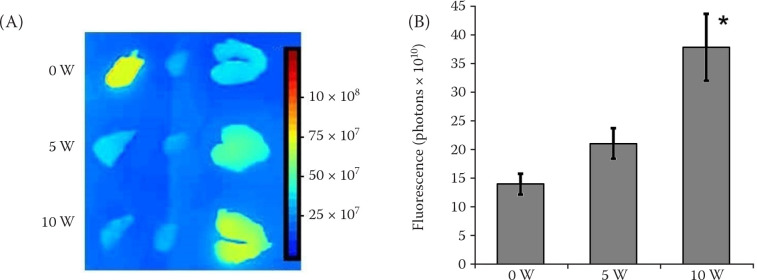
Quantification of exosome deposition in the lung after the application of different sono-stimulation variants (0 W, 5 W, and 10 W) Statistical significance was defined as *P* < 0.05. *Used for significant difference between rats stimulated by 10 W sono and rats were not stimulated (0 W) (A) Representative scans of liver, heart, and lungs from the various experimental variants. The 0 W variant shows high exosome dissipation outside the lungs, whereas the 5 W and 10 W variants show greater accumulation in the lungs after 60 min of circulation. The detection of overall organ fluorescence and export of pseudocolour images were performed using the Bruker Xtreme II system (units: photons/second × mm^2^). (B) Quantification of exosome fluorescence in the lung deposits, recalculated by Xtreme II software (photons per 1 cm^2^ of lung). The results represent average values from four scans per experimental group (two animals, each with dorsal and ventral organ scans)

An additional seven animals (definition of groups in Figure 3) were used to evaluate (*i*) the effective ultrasound penetration into lung tissue, (*ii*) the inflow time of the “first pass” through the lungs, and (*iii*) the hypothesis that the ratio of sono-activated redistribution between the lungs and other organs differs significantly compared with redistribution after stimulation at a lower or zero intensity. These three experiments were arranged in the following sequence:

- The basic hypothesis concerning effective ultrasound penetration through chest tissue to lung tissue was evaluated using the administration of the contrast agent SonoVue into the circulating blood from the tail to the lung. High-tech imaging was performed with an Aixplorer Mach 30 sonograph (Supersonic Imagine, Aix-en-Provence, France), which applied 1-MHz pulses for SonoVue cavitation. The SonoVue sono-reflectance signal was detected precisely at the time of passage through the lungs.
- The detection of the maximal sono-reflectance signal was used for precise determination of the time T_F_ (time of the “first pass” after injection) (see Figure 1B for details).
- The exosome distribution after sono-stimulation of the lung capillaries was analysed. The method was based on the injection of PKH26-labelled exosomes (Sigma-Aldrich, Darmstadt, country; PKH-26 staining performed according to the manufacturer’s instructions). Following exosome injection, a 60-minute interval followed during which the rat remained immobile under inhalation anaesthesia. After this period, the rats were sacrificed, and their organs were extracted. The fluorescence deposition in the organs was quantified using the Bruker Xtreme II system (see Figure 3 for details; excitation: 570 nm, emission: 595 nm).

### Technical details of the stimulator

Sono-stimulation for group A3 was applied using a custom-built ultrasound generator and contact head (experimental device SKP1000, developed by the Czech Technical University in Prague, Czech Republic) operating at 1 MHz. The device included an application head with a circular contact surface with a 6 cm radius. The method used for establishing contact with the animal is shown in Figure 2B.

### Histological analysis of lung tissue

Sampling of lung and other organs (for analysis of exosome distribution and fibrosis scoring) was carried out on day 15 (in two randomly selected animals from each experimental group) and on the terminal day 21 (the remaining four to six surviving animals in each experimental group). The animals were immobilised with 3% isoflurane anaesthesia and euthanised with a guillotine (Harvard Apparatus, Holliston, USA). The animals’ chests were quickly opened, and the tissues were fixed in 4% paraformaldehyde for 24 h, embedded in paraffin, and sectioned at a thickness of 4 μm. The sections were stained with haematoxylin and eosin (H&E) and Goldner’s trichrome. Microscopic observation was performed using an Olympus BX63 microscope. Fibrotic changes were evaluated under light microscopy in ten randomly selected fields in three transverse planes from the basal part of the left lung lobe. The Ashcroft scoring system was used to assess the severity of fibrosis in lung sections from different animal groups (Ashcroft et al. 1988).

### Statistical analysis

Animal studies and cellular data are reported as the mean ± standard deviation (SD) of at least three independent experiments. The comparisons between the groups were performed using MATLAB (MathWorks, USA). The data were analysed using the one-way analysis of variance (ANOVA), followed by post hoc *t*-tests. Statistical significance was defined as *P* < 0.05.

## RESULTS

### Exosome production and increased bioactive miRNA content

**Figure 4:**
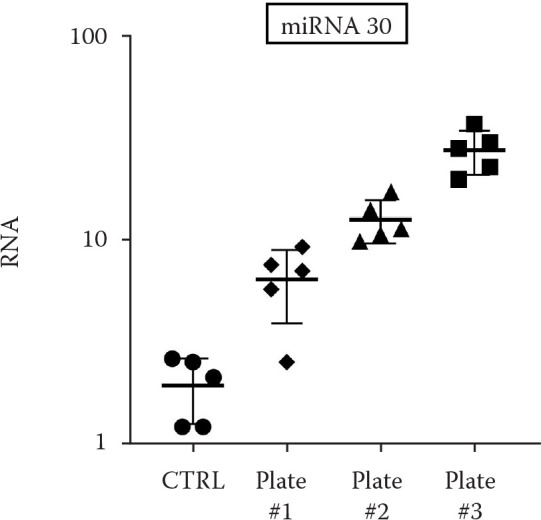
Quantification of miRNA-30 in exosomes from three different MSC cultures after 72 h of incubation Plate #1 was incubated without biophysical stimulation; Plate #2 received only electromagnetic stimulation; Plate #3 was subjected to electromagnetic stimulation combined with additional hypoxia and ultrasound stimulus (0.2 W/cm^2^, 1 MHz) (details of stimulus timing are shown in Figure 2). The CTRL plate was an additional control variant of the cell culture without trehalose and without any biophysical stimulation. Relative gene expression was calculated using the 2^–ΔΔCt^ method

Absolute concentrations of total miRNA and miRNA-30b were quantified per microlitre of culture medium. Figure 4 shows an increase in the miRNA-30b levels in exosomes derived from conditioned medium following electromagnetic stimulation (Plate #2) and combined stimulation (Plate #3).

### Quantification of fibrotic changes in lung tissue

**Figure 5:**
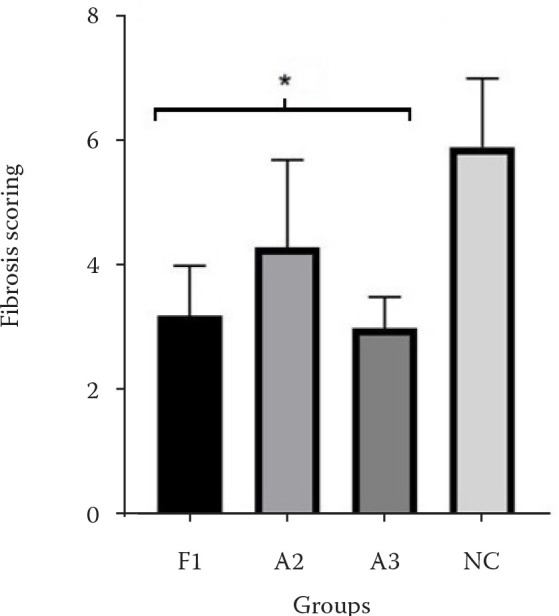
Fibrosis scores for the different experimental groups *Groups F1, A2, and A3 displayed significantly decreased values compared with the control group NC. Statistical significance was defined as *P* < 0.05 (details of the statistical methods in the chapter Material and Methods)

Table 2 presents data obtained from the quantification of histological images at day 21. The exosomes from Plate #3 demonstrated a pronounced antifibrotic effect, comparable to that of the tamoxifen + metformin drug combination. However, this effect was dependent on the application of the ultrasound pulse, which probably played a key role in facilitating exosome diffusion from the capillaries into the lung tissue during first-pass circulation through the pulmonary vasculature. The statistical output for the fibrosis scores at day 21 is presented in Figure 5. A similar statistical analysis was performed using the histological evaluation of the lungs at day 15; however, the differences between the groups at this earlier time point were noticeably smaller (data not shown).

**Table 2:** Demonstration of the different development of fibrosis after different curing methods

Group	Injection	Histological micrograph of lung tissue (Goldner‘s Green Trichrome staining variant)	Number of deaths during the experiment	Fibrosis scores
F1	tamoxifen metformin	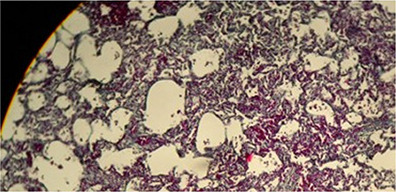	20%	3.2 ± 0.8
A2	EXO only	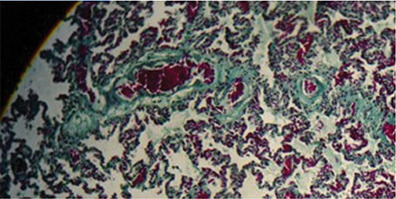	20%	4.3 ± 1.4
A3	EXO + SONO (10 W/cm^2^)	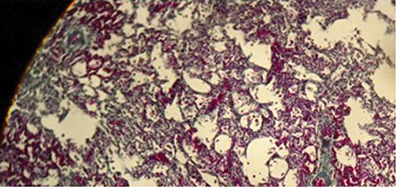	10%	3.1 ± 0.5
NC	PBS only	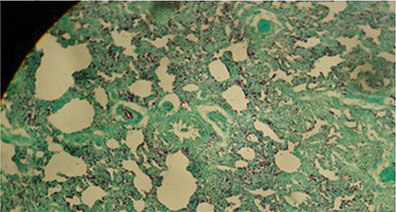	30%	5.6 ± 1.1

### Results of SonoVue calibration and exosome tracking

The results from the additional seven animals confirmed the penetration of ultrasound into lung tissue, quantified the inflow time of first-pass circulation through the lungs, and demonstrated that the ratio of sono-activated exosomes distributed between the lungs and other organs depended on the intensity of the ultrasound. SonoVue-based imaging showed a first-pass inflow time of T_influx_ = T_0_ – T_F_ = 4.5 ± 0.8 s (mean from four animals). The dose with added SonoVue produced a strong contrast, confirming both the transmission of the ultrasound pulse through the skin and chest and the suitability of the ultrasound parameters used. Figure 3 provides a clear illustration of this biophysical phenomenon: higher ultrasound intensity correlated with increased exosome accumulation in the lungs. The increased pulmonary capture is consistent with the hypothesis involving capillary wall undulation, as described previously (see Figure 2B). The selected ultrasound intensity was 10 W/cm^2^. The commonly accepted safety limit for clinical ultrasound applications is 3 W/cm^2^. Although the effective intensity in our study was 10 W/cm^2^, the risk of tissue damage was considered minimal, as ultrasound was applied only within a 200-ms window at the T_influx_ time point.

## DISCUSSION

Pulmonary fibrosis remains a significant clinical challenge and is the focus of ongoing research. To date, no pharmacological agent has effectively prevented pulmonary fibrosis with minimal adverse effects (Henna and Minna 2020; Agudelo et al. 2022; Pak et al. 2024). Pulmonary fibrosis is not only a consequence of the recent COVID-19 pandemic, but this degenerative condition is also increasingly diagnosed in veterinary medicine. This may be associated with industrial air pollution, radon exposure in remote areas, and a higher incidence of new viral respiratory diseases in recent decades (Henna and Minna 2020; Agudelo et al. 2022; Pak et al. 2024).

The diffusion of intravenously administered liposomes from the pulmonary capillaries into the lung interstitium can significantly influence the exposure of interstitial cells to bioactive signals and the speed of onset of the antifibrotic effect. For particles of a comparable size, the diffusion kinetics from circulation into tissue are typically modulated by chemical enhancers (Szabova et al. 2023). In this study, we investigated a physical stimulus – ultrasound. Ultrasound offers a major advantage over chemical enhancers: precise localisation to the target region and a rapid on/off effect. Our results demonstrated that ultrasound significantly increased capillary permeability, and the ratio of exosomes captured in the lungs compared with the liver was markedly higher (Figure 3). Histological analysis also confirmed the safety of direct intravenous exosome administration. Exosomes are smaller than circulating MSCs and erythrocytes, making vascular obstruction or clot formation less likely. In our histological analysis, no visible clots or pathological changes associated with exosome accumulation were observed in the examined regions of the lungs, heart, or liver.

Treatment with exosomes derived from stimulated MSCs significantly reduced fibrotic areas in lung tissue (Table 2 and Figure 5). Similarly, Western blot analysis revealed decreased expression of the fibrosis-related factor α-SMA in lung tissue, and the levels of the proinflammatory cytokine IL-1 were also reduced compared with animals treated with a bolus of non-activated exosomes or exosomes without lung-targeted sono-pulse (data not shown; a comprehensive biochemical analysis of the lungs is being prepared for publication).

Rats are a widely used model for studying pulmonary fibrosis. There is evidence that exosomes derived from MSCs contribute to reversing epithelial-mesenchymal transition (EMT), a process of cellular trans-differentiation in which epithelial cells progressively lose cell connectivity and polarity and acquire mesenchymal characteristics, thereby mitigating lung fibrosis (Zhang et al. 2023; Chang et al. 2024). Furthermore, the effects of exosomes, whether beneficial or detrimental, largely depend on their molecular cargo, among which microRNAs (miRNAs) play a crucial role (Yu et al. 2014; Guo et al. 2023; Xourafa et al. 2024).

Our study primarily described the therapeutic effect on lung tissue from a phenomenological perspective. In several previous studies, MSCs have been activated or stimulated using genetic or chemical methods (Tyciakova et al. 2015; Siciliano et al. 2016).

In contrast, the present study employed biophysical stimulation using electromagnetic and ultrasound applicators. The parameters for these applicators were selected based on previous evaluations of biophysical effects on cells and tissues (Chen et al. 2013; Prucha et al. 2019), which identified safe exposure levels for the physical fields used. Further studies are required to elucidate the underlying mechanisms through detailed microscopy and temporal profiling of proinflammatory and profibrotic markers and growth factors. High-resolution intravital microscopy may provide valuable insights into the mechanism of rapid endothelial regeneration and assist in optimising the frequency and power of the ultrasound pulse or its combination with magneto- or chemo-attractive forces to enhance interstitial diffusion of exosomes and other regenerative factors.
